# The Effect of Electroacupuncture with Different Frequencies on Muscle Oxygenation in Humans

**DOI:** 10.1155/2015/620785

**Published:** 2015-03-02

**Authors:** Kenichi Kimura, Takayoshi Ryujin, Makoto Uno, Ikuro Wakayama

**Affiliations:** Department of Health Sciences, Kansai University of Health Sciences, Osaka 590-0482, Japan

## Abstract

The aim of the present study was to investigate the effect of electroacupuncture (EA) with different frequencies on muscle oxygenation in humans. The subjects were 8 healthy male volunteers. Muscle oxygenation was measured using near-infrared spectroscopy (NIRS). Blood pressure (BP) and heart rate (HR) were monitored simultaneously. After baseline recording, EA was given for 15 min and recovery was measured for 20 minutes. The procedure of EA at 1 Hz, at 20 Hz, and at control followed in the same subjects. Tissue oxygenation index (TOI) decreased during EA at 20 Hz (*P* < 0.05) and increased during the recovery period. Normalized tissue hemoglobin index (nTHI) also decreased during EA at 20 Hz and increased during the recovery period (*P* < 0.05), whereas TOI and nTHI in the EA at 1 Hz did not change significantly throughout the experiment. The peak TOI and nTHI values at 20 Hz during the recovery period were higher than the values at 1 Hz and in the control (*P* < 0.05). BP and HR remained constant. These data suggest that the supply of oxygen to muscle decreased during EA at 20 Hz and increased after EA at 20 Hz, without any changes in HR and BP.

## 1. Introduction

It is well known that electroacupuncture (EA) has clinical effects on pain relief in many disorders. As for the mechanism underlying these effects, it is thought to be effective in improving local blood flow, and improvements in local circulation may flush out algesic or sensitizing substances leading to pain relief. Therefore, EA has been widely used for musculoskeletal impairments. Different from transcutaneous electrical nerve stimulation (TENS), which stimulates the skin surface, EA does not require consideration of the high impedance of skin as it involves electrical stimulation of nerve or muscle through inserted acupuncture needles in vivo as electrodes. Thus, it can be used with a level of stimulus output lower than that in TENS. Furthermore, EA has the benefit of enabling alteration of the stimulus conditions quantitatively, such as in terms of frequency, duration, and stimulus intensity. However, the optimal stimulus conditions of EA for clinical effects have yet to be standardized. It is clinically important to clarify the effects of EA with different frequencies on muscle oxygenation. To the current authors' knowledge, the effects of EA with different frequencies on muscle oxygenation have not been reported.

We used near-infrared spectroscopy (NIRS) to measure muscle oxygenation in the present study. NIRS has been used in recent years to measure muscle oxygenation noninvasively in humans [1, 2]. However, its usefulness is limited due to the influence of skin blood flow (SBF) on the NIRS variables. Recent studies have indicated the influence of SBF on NIRS measurements in skeletal muscle [3–5]. Even small increases in SBF may affect NIRS light absorption and potentially confound the interpretation of the underlying muscle oxygenation measurement. In this respect, spatially resolved spectroscopy (SRS) was developed to minimize the influence of SBF when assessing changes in deep tissue oxygenation [6, 7]. Therefore, SRS-based NIRS was used to measure muscle oxygenation in the present study. The objective of the present study was to investigate the effect of EA with different frequencies on muscle oxygenation in humans.

## 2. Methods

### 2.1. Subjects

Eight healthy male subjects participated in this study. Their mean age, height, and weight were 21.1 ± 0.3 yrs, 1.71 ± 0.2 m, and 58.4 ± 2.9 kg, respectively. All subjects were normotensive, taking no medications, and free of cardiovascular or neuromuscular diseases, based on medical history and physical examination. The study was approved by the Institutional Review Board of Kansai University of Health Sciences in terms of all protocols and informed consent documents, in accordance with the ethical standards laid down in the 2013 Declaration of Helsinki.

### 2.2. Measurement of Muscle Oxygenation

**Figure 1:**
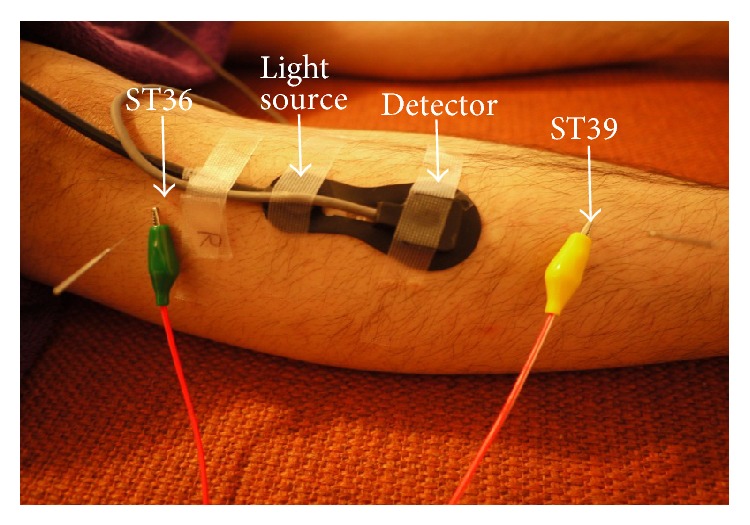
Measurement site and electroacupuncture (EA) stimulation point.

Muscle oxygenation was measured continuously using a NIRS system (NIRO-200, Hamamatsu Photonics, Hamamatsu, Japan) in the present study. The NIRO-200 is a noninvasive monitor that employs laser diodes emitting light at different wavelengths of 775, 810, and 850 nm and corrects scattered light signals by two closely placed detectors. The light paths leading from the emitter to the different detectors share a common part, the one related to superficial tissues, while the path length in the deep tissues is longer for the detectors at greater distance. Thus, by analyzing the differential signal collected by the detectors, SRS variables are intrinsically more sensitive to the changes occurring in deep tissue layers than variables computed according to the standard modified Beer-Lambert law (MBL). Although MBL-based parameters were shown to be easily affected by the changes in cutaneous perfusion, SRS-based parameters were reported to minimize the contribution of superficial tissue layers in both cerebral monitoring and muscle monitoring [7–9]. The SRS relies on measurement of the attenuation gradient as a function of source-detector separation. With the use of a modified diffusion equation, a product of the absorption and scattering coefficients is calculated [10]. SRS measures tissue oxygenation using the tissue oxygenation index (TOI) and the normalized tissue hemoglobin index (nTHI). TOI is the change in concentration of oxygenated to total tissue hemoglobin and can be expressed as follows: TOI = 100 × oxyhemoglobin (O_2_Hb)/O_2_Hb + deoxyhemoglobin (HHb), expressed in percent, and total hemoglobin (O_2_Hb + HHb) concentration by the nTHI, expressed in arbitrary units (a.u.) [7]. These two parameters allow the assessment of relative changes in tissue oxygenation and tissue blood volume. The probe was attached on the skin over the middle of the left tibialis anterior muscle (Figure 1). NIRS optodes were set at a distance of 4 cm using a rubber holder secured to the skin with double-sided adhesive tape and further stabilized with a crepe bandage around the lower leg. The light in the laboratory was turned off and external light was blocked with a curtain throughout the experiment in order to avoid its influence on NIRS measurement.

### 2.3. Electroacupuncture

Acupuncture needles (0.20 × 40 mm; Seirin Kasei Co., Shizuoka, Japan) were inserted into ST36 and ST39 vertically on the left tibialis anterior muscle at a depth of approximately 10 mm (Figure 1). The ST36 point was chosen for this study since it is commonly considered to improve peripheral circulatory disturbance and digestive functions in traditional Chinese medicine [11]. The needle inserted at ST36 was connected to the cathode and the needle at ST39 was connected to the anode (Figure 1). Electrical stimulation was delivered for 15 min with a square wave of duration of 0.2 ms at 1 Hz or 20 Hz using an electrical stimulator (Ohm Pulser, LFP-4000A, Zen Iryoki, Fukuoka, Japan). The stimulus intensity was adjusted to produce local muscle twitch, as strong as possible, without pain or discomfort. As a control, subjects were placed on a bed for 40 min without EA. Acupuncture was performed by a certified acupuncturist with more than 10 years of experience.

### 2.4. Experimental Protocol

Upon entering the laboratory, each subject rested comfortably in a supine position on a bed in the laboratory with the temperature controlled at 24°C. After recording for 5 min at rest (baseline period), EA was applied for 15 min while monitoring continued (EA period). After the cessation of EA, recovery measurements were made for 20 min (recovery period). Thus, the muscle oxygenation recording was performed continuously for 40 min. Simultaneously, heart rate was obtained from the* R*-*R* interval of the electrocardiogram (ECG) and arterial blood pressure was measured noninvasively with a finger arterial blood pressure monitor (MUB-101, MEDISENS, Tokyo, Japan). The mean arterial pressure (MAP) was calculated from the diastolic blood pressure (DBP) and systolic blood pressure (SBP) as MAP = DBP + (SBP − DBP)/3. MAP and HR were averaged every 2 min for analysis, except during the first minute of EA. Each protocol (control, 1 Hz, and 20 Hz) was conducted at random on a separate day in the same group of nine normal subjects. All parameters were recorded in the same manner in each protocol.

## 3. Data Analysis

Data were continuously obtained and digitized at a sampling rate of 1 kHz (Power Lab 8/30; AD Instruments, Sydney, Australia). TOI and nTHI were averaged at 1-minute periods for analysis. Repeated measures two-way analysis of variance (ANOVA) was used to detect significant interaction for all variables between frequencies (control, 1 Hz, and 20 Hz). For one-way ANOVA,* Dunnett's test* was used to evaluate the serial changes over time in each parameter compared with the baseline values. Peak TOI and nTHI values during recovery periods were compared between each frequency using Tukey's post hoc analysis. Values are the mean ± SEM. The significance of differences was accepted at *P* < 0.05.

## 4. Results

**Figure 2:**
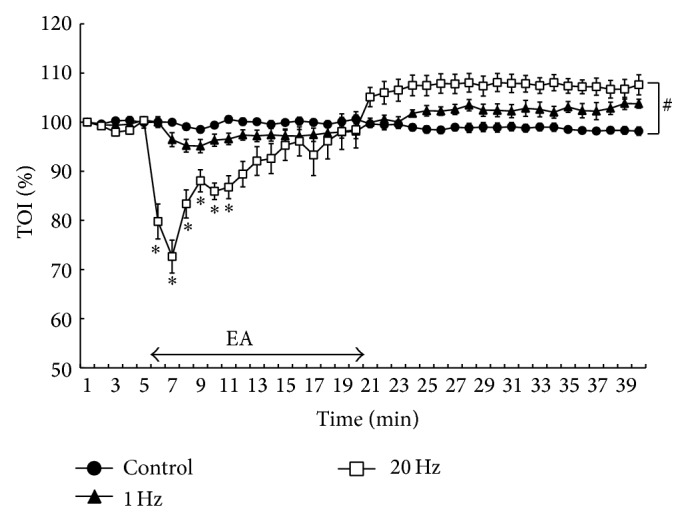
Percent changes in tissue oxygenation index (TOI) during electroacupuncture (EA) stimulation. ^*^ *P* < 0.05 versus baseline values. ^#^Significant difference from control and 1 Hz (*P* < 0.05). Values are mean ± SEM.

**Figure 3:**
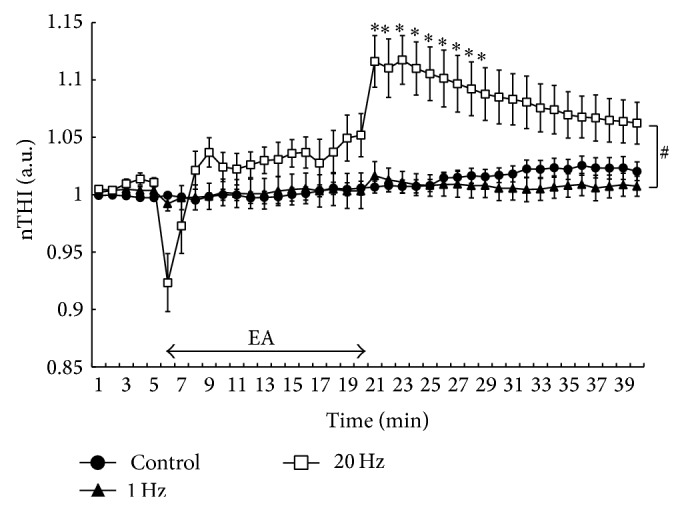
Serial changes in normalized tissue hemoglobin index (nTHI) values during electroacupuncture (EA) stimulation. ^*^ *P* < 0.05 versus baseline values. ^#^Significant difference from control and 1 Hz (*P* < 0.05). Values are mean ± SEM.

Figure 2 shows the percent changes in TOI values for each protocol throughout the experiment. TOI and nTHI did not appear to be changed by EA at 1 Hz or in the control throughout the experiment. During EA at 20 Hz, the TOI values decreased significantly (*P* < 0.05; Figure 2) and increased immediately after EA at 20 Hz compared with the baseline values. Furthermore, there was a significant interaction for TOI between 20 Hz and 1 Hz or the control (*P* < 0.05; Figure 2).  Figure 3 shows the changes in nTHI in the baseline period, during EA, and in the recovery period. The nTHI values decreased transiently and returned to the baseline during EA at 20 Hz and increased markedly after EA at 20 Hz compared with the baseline (*P* < 0.05; Figure 3). There was a significant interaction for nTHI between 20 Hz and 1 Hz or the control (*P* < 0.05; Figure 3).

**Figure 4:**
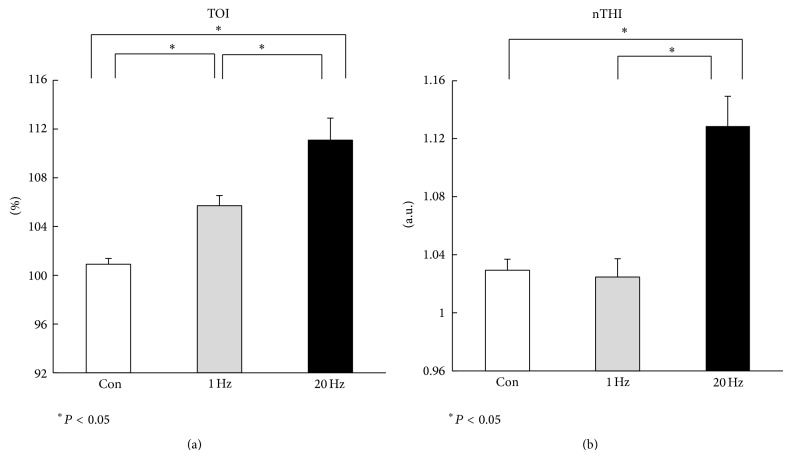
(a) Peak tissue oxygenation index (TOI) and (b) normalized tissue hemoglobin index (nTHI) values during recovery period at control, 1 Hz, and 20 Hz. Peak TOI and nTHI values were significantly higher at 20 Hz, compared with those at the control and 1 Hz. ∗Significant difference from control or 1 Hz (*P* < 0.05). There was significant difference in peak TOI values between control and 1 Hz (*P* < 0.05). There was no difference in peak nTHI values between control and 1 Hz. Values are mean ± SEM.

**Figure 5:**
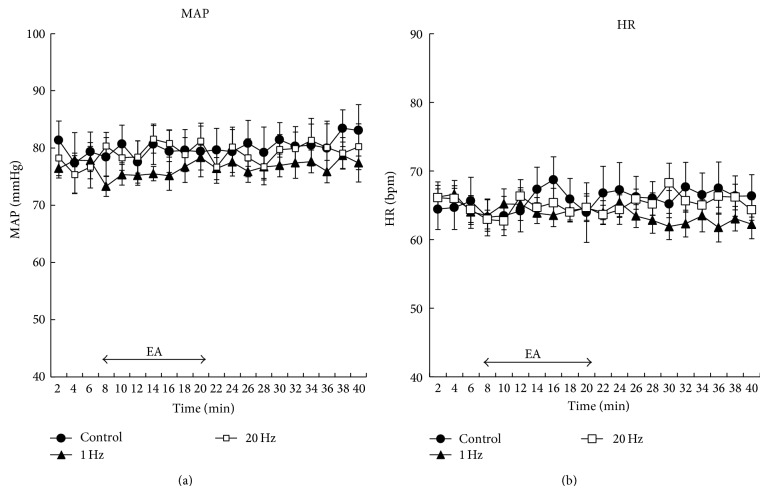
Serial changes in (a) mean arterial pressure (MAP) and (b) heart rate (HR) at control, 1 Hz, and 20 Hz. MAP and HR were averaged every 2 minutes. Symbols represent 2-minute average values. There was no significant change in MAP and HR compared with baseline values. Values are mean ± SEM.

At baseline, TOI and nTHI values were similar between each protocol (*P* > 0.05). However, there was a significant difference in peak TOI values during the recovery period between the values at 20 Hz (111.07 ± 1.81%; *P* < 0.05; Figure 4(a)) and the values at 1 Hz (105.70 ± 0.84%) or the control values (100.90 ± 0.48%). For nTHI, peak values at 20 Hz during the recovery period were significantly higher (1.12 ± 0.02 a.u.; *P* < 0.05; Figure 4(b)) relative to the values at 1 Hz (1.02 ± 0.01 a.u.) or the values in the control (1.03 ± 0.01 a.u.). Average HR and MAP remained unchanged throughout each protocol (*P* > 0.05, Figure 5). In addition, none of the subjects reported feeling any sensation of pain during EA stimulation.

## 5. Discussion

The results of the present study demonstrated decreases in TOI and nTHI during EA and increases in them after EA. In addition, EA at 20 Hz more significantly decreased TOI and nTHI during EA and increased these after EA than EA at 1 Hz. EA is generally employed for acupuncture, but the effects of EA frequency on muscle oxygen dynamics remain unclear. The results of the present study demonstrated that the effects of EA on muscle oxygen dynamics vary with frequency.

Manual acupuncture improves local muscle oxygen dynamics [12–14], but whether EA has similar effects remains unclear. Oxygen supply to muscle tissue is promoted as muscle blood flow increases and reduces as it decreases. Several reports have been published on the improvement of muscle blood flow by acupuncture. Jansen et al. [15] reported that EA administration to the flap increased the blood flow to the same extent as calcitonin gene-related peptide (CGRP) in anesthetized rats. Sato et al. [16] reported that muscle blood flow was increased by electrically stimulating the dorsal roots and decreased by administering a CGRP receptor blocker in anesthetized rats, suggesting the involvement of CGRP in the increased muscle blood flow. On the other hand, the involvement of NO released from vascular endothelial cells has attracted attention. Loaiza et al. [17] reported that NO was involved in locally increased blood flow with EA. Tsuchiya et al. [18] reported that blood NO concentration and blood flow increased in the upper arm that underwent acupuncture; these showed a significant positive correlation. Also we reported that increased blood flow by acupuncture was suppressed by administering a NO synthase inhibitor [19]. Thus, the production of vasodilatory substances, such as CGRP and NO, is involved in the improvement of local blood flow by manual acupuncture or EA.

Besides the above-mentioned vasodilatory mechanisms, the muscle relaxation occurring at the end of EA may have contributed to the ensuing hyperemia. The decrease in TOI and nTHI during EA suggests that blood flow is influenced by intramuscular pressure increased by muscle tension during muscle contraction. Intramuscular pressure tends to increase as the muscle thickness increases. In particular, the tibialis anterior muscle is thick and covered with less elastic fascia muscle, increasing intramuscular pressure [20]. The muscular vessels were subjected to mechanical pressure due to muscle contraction during EA, reducing blood flow and decreasing TOI and nTHI.

Furthermore, the vascular smooth muscles in skeletal muscle are regulated by efferent adrenergic muscle sympathetic nerve activity (MSNA). Knardahl et al. [21] recorded MSNA using the peroneal nerve of a healthy individual and performed EA between LI4 and LI11. As a result, the number of bursts significantly increased after EA compared with those before EA. The decreased TOI and nTHI during EA may have been caused by the decreased muscle blood flow due to the enhanced MSNA. Specifically, the excitement of mechanoreceptors due to muscle contractions during EA enhances MSNA through the cardiovascular center and reflexively decreases muscle blood flow, reducing oxygen supply [22]. However, MSNA enhancement does not necessarily reduce blood flow in the skeletal muscles [23]. This is explained by vasodilation due to metabolites generated by muscle contraction, which antagonizes vasoconstriction due to MSNA enhancement. Slight MSNA enhancement may insufficiently reduce blood flow in the skeletal muscles. In addition, in the present study, TOI and nTHI decreased only transiently during EA, suggesting that vasodilatation due to metabolites generated by muscle contraction antagonizes vasoconstriction due to MSNA enhancement.

TOI and nTHI during EA at 20 Hz more significantly decreased than those during EA at 1 Hz, suggesting that muscle contraction during EA at 1 Hz was increased by muscle contraction during EA at 20 Hz, significantly increasing muscle tension. Muscle tension increased during EA at 20 Hz, mechanically compressing the muscle blood vessels more strongly than at 1 Hz to reduce blood flow. TOI and nTHI more significantly increased after EA at 20 Hz than after EA at 1 Hz, suggesting that blood flow more significantly increases after more intensive muscle contraction [24]. Thus, muscle tension became more intense and more significantly increased blood flow during EA at 20 Hz than EA at 1 Hz. Increased shear stress on the vessel wall, caused by increased blood flow, stimulates vascular endothelial cells to release NO, further increasing blood flow [25].

Finally, local muscle oxygen dynamics was changed by EA at 20 Hz, although neither heart rate nor average blood pressure significantly changed. EA reduced blood flow in the internal organs through the sympathetic nerve in anesthetized rats, elevating blood pressure and passively increasing muscle blood flow [26]. However, in the present study, EA at both 1 and 20 Hz caused no change in systemic blood pressure, suggesting that changes in local muscle oxygen dynamics are localized independently of blood pressure.

## 6. Conclusions

In the present study, oxygen dynamics in muscle tissue was improved using NIRS after EA at 20 Hz, and the oxygen supply to muscle tissue did not change significantly after EA at 1 Hz. Thus, the frequency of 20 Hz is recommended for muscle fatigue and muscle pain in acupuncture treatment, in order to perform EA to facilitate the blood flow and oxygen supply in affected muscles. Stimulus conditions, including duration, waveform, should be further investigated in the future.

## References

[1] Boushel R., Piantadosi C. A. (2000). Near-infrared spectroscopy for monitoring muscle oxygenation. *Acta Physiologica Scandinavica*.

[2] Grassi B., Pogliaghi S., Rampichini S. (2003). Muscle oxygenation and pulmonary gas exchange kinetics during cycling exercise on-transitions in humans. *Journal of Applied Physiology*.

[3] Buono M. J., Miller P. W., Hom C., Pozos R. S., Kolkhorst F. W. (2005). Skin blood flow affects *in vivo* near-infrared spectroscopy measurements in human skeletal muscle. *Japanese Journal of Physiology*.

[4] Davis S. L., Fadel P. J., Cui J., Thomas G. D., Crandall C. G. (2006). Skin blood flow influences near-infrared spectroscopy-derived measurements of tissue oxygenation during heat stress. *Journal of Applied Physiology*.

[5] Tew G. A., Ruddock A. D., Saxton J. M. (2010). Skin blood flow differentially affects near-infrared spectroscopy-derived measures of muscle oxygen saturation and blood volume at rest and during dynamic leg exercise. *European Journal of Applied Physiology*.

[6] Matcher S. J., Kirkpatrick P. J., Nahid K., Cope M., Delpy D. T. Absolute quantification methods in tissue near-infrared spectroscopy.

[7] Al-Rawi P. G., Smielewski P., Kirkpatrick P. J. (2001). Evaluation of a near-infrared spectrometer (NIRO 300) for the detection of intracranial oxygenation changes in the adult head. *Stroke*.

[8] Canova D., Roatta S., Bosone D., Micieli G. (2011). Inconsistent detection of changes in cerebral blood volume by near infrared spectroscopy in standard clinical tests. *Journal of Applied Physiology*.

[9] Messere A., Roatta S. (2013). Influence of cutaneous and muscular circulation on spatially resolved versus standard Beer–Lambert near-infrared spectroscopy. *Physiological Reports*.

[10] Suzuki S., Takasaki S., Ozaki T., Kobayashi Y. Tissue oxygenation monitor using NIR spatially resolved spectroscopy.

[11] Cheng Y., Huang X., Jia W. (1999). *Chinese Acupuncture and Moxibustion*.

[12] Jimbo S., Atsuta Y., Kobayashi T., Matsuno T. (2008). Effects of dry needling at tender points for neck pain (Japanese: Katakori): near-infrared spectroscopy for monitoring muscular oxygenation of the trapezius. *The Journal of Orthopaedic Science*.

[13] Ohkubo M., Niwayama M., Murase N. (2009). Local increase in trapezius muscle oxygenation during and after acupuncture. *Dynamic Medicine*.

[14] Cagnie B., Barbe T., De Ridder E., Van Oosterwijck J., Cools A., Danneels L. (2012). The influence of dry needling of the trapezius muscle on muscle blood flow and oxygenation. *Journal of Manipulative and Physiological Therapeutics*.

[15] Jansen G., Lundeberg T., Kjartansson J., Samuelson U. E. (1989). Acupuncture and sensory neuropeptides increase cutaneous blood flow in rats. *Neuroscience Letters*.

[16] Sato A., Sato Y., Shimura M., Uchida S. (2000). Calcitonin gene-related peptide produces skeletal muscle vasodilation following antidromic stimulation of unmyelinated afferents in the dorsal root in rats. *Neuroscience Letters*.

[17] Loaiza L. A., Yamaguchi S., Ito M., Ohshima N. (2002). Electro-acupuncture stimulation to muscle afferents in anesthetized rats modulates the blood flow to the knee joint through autonomic reflexes and nitric oxide. *Autonomic Neuroscience*.

[18] Tsuchiya M., Sato E. F., Inoue M., Asada A. (2007). Acupuncture enhances generation of nitric oxide and increases local circulation. *Anesthesia & Analgesia*.

[19] Kimura K., Takeuchi H., Yuri K., Wakayama I. (2013). Effects of nitric oxide synthase inhibition on cutaneous vasodilation in response to acupuncture stimulation in humans. *Acupuncture in Medicine*.

[20] Pedowitz R. A., Hargens A. R., Mubarak S. J., Gershuni D. H. (1990). Modified criteria for the objective diagnosis of chronic compartment syndrome of the leg. *The American Journal of Sports Medicine*.

[21] Knardahl S., Elam M., Olausson B., Wallin B. G. (1998). Sympathetic nerve activity after acupuncture in humans. *Pain*.

[22] Rowell L. B. (1994). *Human Cardiovascular Control*.

[23] Rådegran G., Saltin B. (1998). Muscle blood flow at onset of dynamic exercise in humans. *American Journal of Physiology—Heart and Circulatory Physiology*.

[24] Kagaya A., Homma S., Kuno M. (1994). Brachial arterial blood flow modification during dynamic handgrip exercise at a frequency of one contraction per second. *Journal of Exercise Science*.

[25] Joyner M. J., Dietz N. M. (1997). Nitric oxide and vasodilation in human limbs. *Journal of Applied Physiology*.

[26] Noguchi E., Ohsawa H., Kobayashi S., Shimura M., Uchida S., Sato Y. (1999). The effect of electro-acupuncture stimulation on the muscle blood flow of the hindlimb in anesthetized rats. *Journal of the Autonomic Nervous System*.

